# miR-34a mimic or pre-mir-34a, which is the better option for cancer therapy? KatoIII as a model to study miRNA action in human gastric cancer cells

**DOI:** 10.1186/s12935-021-01872-5

**Published:** 2021-03-19

**Authors:** Narjes Jafari, Saeid Abediankenari, Hadi Hossein-Nataj

**Affiliations:** 1 Immunogenetics Research Center, Faculty of Medicine, Mazandaran University of Medical Sciences, Sari, Iran; 2 Department of Immunology, Faculty of Medicine, Mazandaran University of Medical Science, Sari, Iran

**Keywords:** MicroRNA-34a, Gastric cancer, Cancer stem cells, Transfection, Precursor miRNA

## Abstract

**Background:**

Aberrantly expressed microRNAs play important roles in gastric tumorigenesis. However, use of miRNAs as a therapeutic option in gastric cancer still remains as a challenging problem.

**Methods:**

We performed transient transfection of miR-34a-5p mimic and stable transfection of pre-mir-34a into KatoIII cells. Then, we evaluated the effect of transfected miRNAs on numerous cellular and molecular processes.

**Results:**

Following transient transfection of miR-34a-5p mimic at 25 nM—a commonly used concentration—into KatoIII cells, inhibition of two target genes expression, namely Notch1 and β-catenin, was not observed, but a non-significant marginal increase of these genes was detected. No changes were detected in the percentage of apoptotic cells as well as in CD44 + and EpCAM + cells after 25 nM miR-34a-5p mimic transfection. Interestingly, stable transfection of pre-mir-34a into KatoIII cells (named as KatoIII-pGFPC1-34a cells) caused a significant repression in β-catenin protein and Notch1 mRNA levels (p < 0.05 and p < 0.01, respectively) relative to equivalent control (KatoIII- pGFPC1-empty cells). The percentage of CD44 + cells in the KatoIII-pGFPC1-34a cells (< 40%) was significantly lower than that in control cells (~ 95%) (p < 0.05). An increase of ~ 3.5% in apoptotic cells and a slower proliferation rate were detected in KatoIII-pGFPC1-34a cells.

**Conclusions:**

Our study revealed that the effect of miR mimic in target gene repression can be dependent to its concentration as well as to the cell type. Meanwhile, our findings further support a regulatory function for pre-miRNAs in target repression and will help to develop effective therapeutic strategies in cancer treatment.

**Supplementary Information:**

The online version contains supplementary material available at 10.1186/s12935-021-01872-5.

## Background

Gastric cancer (GC) is still a major cause of cancer-related mortality worldwide, with poor prognosis, high recurrence and low survival rates for patients with advanced disease. Despite overall advancements in conventional strategies, surgery, radiotherapy and chemotherapy, the treatment of GC patients still remains a challenging problem [1–3]. Since multiple genetic dysregulations may be implicated in gastric tumorigenesis [4], identification of a pivotal regulatory molecule through profound studies may be helpful for development of effective therapies.

microRNAs (miRNAs, miRs) are small non-coding regulatory RNA molecules that play important roles in various biological processes. One miRNA could target many mRNAs, and most mammalian mRNAs are conserved targets of miRNAs [5–7]. At least three RNA species, primary miRNA (pri-miRNA), precursor miRNA (pre-miRNA), and mature miRNA, are generated from miRNA genes during transcription and sequential processing steps by Drosha and Dicer ribonucleases. Mature miRNAs are incorporated into RNA-induced silencing complexes (RISC) and target cognate mRNAs. Then, they induce translational repression or degradation of mRNAs primarily by binding to one or more target sites in the 3ʹ untranslated region (UTR) of mRNA transcript [6, 8, 9]. All other RNAs produced from miRNA genes (pri-miRNAs and pre-miRNAs) have predominantly been considered to be transitory intermediates with no direct activity in target gene regulation. But, some studies suggested a possible regulatory function of pri and/or pre-miRNAs in target recognition and repression [10, 11].

Numerous studies have revealed the remarkable variations in miRNA expression profiles of cancer cells in comparison with the normal cells and suggested a correlation between aberrantly expressed miRNAs and cancer cell functions, including proliferation, apoptosis, migration, and invasion. On the basis of their altered expression and functions, a few miRNAs have been classified as oncogenes or tumor suppressor genes [12–14].

Previous studies have demonstrated that microRNA-34a-5p (miR-34a-5p) is frequently downregulated or absent in several cancers, including GC, and acts as tumor suppressor with contribution in different cellular processes such as apoptosis and cell cycle arrest [15].

The existing data also suggest that miRNAs might play important roles in function and the stemness maintenance of the cancer stem cells (CSCs)—a small subpopulation of cells in tumors that was known to be responsible for tumor initiation, progression, metastasis, recurrence, and resistance to chemo radiotherapy [6, 16]. However, to date, little is known about the biological and molecular mechanism by which miRNAs regulate CSCs.

In numerous studies, CD44 and EpCAM (epithelial cell adhesion molecule) have been identified as surface markers of gastric CSCs (GCSCs) and used to isolate, enrich and characterize the GCSCs from cell lines and GC tissues [17–19].

Most target genes of miR-34a are frequently up-regulated in human cancers and act as oncogenes. Importantly, among these target genes, several are associated with critical signaling pathways of stem cell, and their aberrant expression can be involved in the self-renewal, survival, stemness maintenance and tumorigenicity of CSCs, such as Notch1,2, β-catenin, and CD44 [15]. Therefore, exploration of miR-34a-5p roles in regulation of various CSCs will be promising to design drugs or effective therapeutic methods to target and eliminate CSCs.

Although enormous advances have been achieved in experimental efforts to restore a loss of miRNAs function in cancer cells through synthetic miRNA mimics transfection, but their efficient functions in cellular physiology remains rather enigmatic and was a challenging problem in some studies.

In this study, with respect to potential tumor-suppressor activity of miR-34a-5p, miR-34a-5p mimic and pre-mir-34a were transfected into KatoIII cells, a GC cell line. Then, we assayed the effects of miR-34a-5p mimic and pre-mir-34a transfection on β-catenin, Notch1, and putative GCSCs markers, namely CD44 and EpCAM, expression level. Furthermore, the possible changes in apoptosis and proliferation rates were also assessed. Our results will provide new insights into possible regulatory effect of mir-34a on multiple oncogenes involved in GCSCs entity and help in the development of effective therapies to treat patients.

## Materials and methods

### Cell lines and culture condition

Human GC epithelial cell lines AGS, KatoIII and normal human bronchus epithelial cell line BEAS-2B were purchased from the cell bank of the Pasteur institute (Iran). The AGS and KatoIII cells were cultured in RPMI-1640 medium (Gibco) supplemented with 10% heat-inactivated fetal bovine serum (FBS) (Biowest) and 1% Penicillin/Streptomycin (Biowest). The BEAS-2B cells were cultured in LHC-9 medium (Life Technologies) supplemented with 10% heat-inactivated FBS and 1% penicillin/streptomycin. All cell lines were incubated in a humidified atmosphere containing 5% CO_2_ at 37 °C.

### RNA extraction, reverse transcription and quantitative real time PCR analysis

Total RNA was extracted from cell lines using Trizol (One step RNA reagent, Biobasic, Cat. No: BS410A) according to the protocol. The concentration of total RNA was determined by a PicoDrop spectrophotometer. All RNA samples were treated with RNase-free DNase I (Thermo Scientific, #EN0521) to remove the possible contamination with DNA.

Complementary DNA (cDNA) synthesis was carried out with 1 µg of DNase I-treated total RNA for each reverse transcription reaction using RevertAid First Strand cDNA synthesis kit (Thermo Scientific, #K1622) following the manufacturer’s protocol. Stem-loop primer was used for the specific cDNA synthesis of miR-34a-5p. Reverse transcription of U6 small nuclear RNA (as internal control for the normalization of miRNA expression) was also performed using a specific primer (Additional file 1: Table S1). Equal volume (1:1) of oligo (dT)_18_ and random hexamer primers was mixed and used for cDNA synthesis from total RNA to quantify the expression of the other genes of interest in this study.

Quantitative real time PCR (qRT-PCR) was performed using SYBR Premix Ex Taq II (Takara, Japan) in Exicycler™ 96 system (Bioneer, Republic of Korea). After an initial denaturation for 2 min at 95 °C, qRT-PCR was followed by 40–45 cycles at 95 °C for 15 s, and at 58 °C for 50 s. The specificity of the amplified products was determined by electrophoresis on 2% agarose gel. U6 snRNA and Glyceraldehyde-3-phosphate-dehydrogenase (GAPDH) were used as internal control for the normalization of miRNA and mRNA expressions, respectively. The relative expression levels of the genes were calculated by the 2^−∆∆Ct^ method and our results were represented as fold changes in expression of the genes of interest after cell transfection with synthetic miR-34a-5p mimic/ pre-mir-34a relative to the control. The sequence of the primers was shown in Additional file 1: Table S1.

### Oligonucleotide transient transfection

KatoIII and BEAS-2B cells were seeded in 24-well plates at a density of 50,000 cells per well the day before transfection. After 24 h incubation, medium in each well was replaced with antibiotic free medium containing 5% FBS. The cells were transfected with miR-34a-5p mimic (5′-UGGCAGUGUCUUAGCUGGUUGU-3′) or miRNA negative control (5′-UUCUCCGAACGUGUCACGUTT-3′) at different concentrations (25 nM, 15 nM, 10 nM and 5 nM) using Lipofectamine® 2000 (Invitrogen) according to the manufacturer’s protocol. At 48 h post- transfection, total RNA was extracted, and qRT–PCR was used to detect changes in miRNAs expression, and to verify the transfection efficiency. All the single stranded RNA oligonucleotides were synthesized by Bioneer Company (Republic of Korea). Double stranded miRNA-34a oligonucleotide (Accutarget Human miRNA mimic, Cat.no. SMM-001) was purchased from the company (Bioneer, Republic of Korea). Silencer® Select GAPDH Positive Control siRNA (cat#4,390,849, Ambion) and Silencer® Select Negative Control siRNA (cat#4,390,843, Ambion) were used as positive and negative controls, respectively, in transient transfection. After siRNA transfection, the GAPDH mRNA was quantified using qRT-PCR method and normalized against β- actin internal control.

### Stable transfection

The hsa-mir-34a DNA with flanking regions (Fig. 4a) was synthesized and cloned into pEGFP-C1 plasmid (4.73 kb) by GeneralBiosystems company (USA). A brief overview of the stable transfection procedure was mentioned as fallows. pEGFP-C1-34a or pEGFP-C1- empty (as a control) was transfected into KatoIII cells using Lipofectamine® 2000 (Invitrogen). After 48 h incubation, the cells were observed under fluorescence microscope and transfection efficiency was examined using green fluorescent protein (GFP) expression. Then, stably transfected cells were selected in medium containing 1000 µg/ml G418 (Melford). The selection medium was changed every 2–3 days. After 4 weeks of incubation in presence of high concentration of G418, the stable cells were passaged and maintained in medium with 50 µg/ml G418 for further analysis such as qRT-PCR and western blot.

### Western blot analysis

The cells were lysed using RIPA buffer (Santa Cruz Biotechnology, sc-24,948) supplemented with protease inhibitor, PMSF (phenylmethylsulfonyl fluoride) and sodium orthovanadate (all Santa Cruz Biotechnology) at a ratio of 150:5:3:3 for 30 min on ice. Cell debris was removed by centrifugation at 10,000×*g* for 10 min at 4 °C. Subsequently, the protein concentration was determined using a Better Bradford Protein Assay Kit (Bio basic INC, Cat. no: SK3041). Equal amounts (60 µg) of each protein extract were separated by 12% SDS-PAGE and subsequently transferred to polyvinylidene fluoride (PVDF) membranes (Amersham, Italy) using Mini Trans-Blot® Cell (Bio Rad). The membranes were blocked with Tween Tris Buffered Saline (TTBS) containing 3% skimmed milk powder for 1 h and incubated with the following primary antibodies diluted at 1:500: mouse anti-β-catenin monoclonal antibody (Santa Cruz Biotechnology, sc-7963), and mouse anti-GAPDH monoclonal antibody (Santa Cruz Biotechnology, sc-365,062) at 4 °C overnight. The membranes were then washed with TTBS for three times and incubated for 2 h at room temperature with a recombinant mouse IgGκ binding protein conjugated to horseradish peroxidase (HRP) (Santa Cruz Biotechnology, sc-516,102), diluted at 1:5000. Following three washes with TTBS, the protein bands were visualized using a Chemiluminescence Detection Kit (Pars tous, B111421) and imaging was performed by G BOX instrument (Syngene company, UK). The protein expression was quantified using ImageJ software (National Institutes of Health, Bethesda, MD, USA), and β-catenin protein level was normalized to GAPDH as internal control.

### Flow cytometry analysis

Transiently transfected KatoIII cells with miR-34a-5p mimic or negative control after 48 h, as well as stably transfected cells were collected by centrifugation at 300×g for 5 min. Antibody staining was performed in 100 µl of phosphate buffer saline (PBS) supplemented with 1% bovine serum albumin (BSA) with the following antibodies: anti-human CD44-FITC (eBioscience, cat.no. 11-0441-81), and anti-human EpCAM-PE (eBioscience, cat.no.12-9326-42). Subsequently, the cells were incubated at 4 °C for 40 min. After being stained, the cells were washed in PBS supplemented with 1% BSA and fixed in 1% formaldehyde. Cells were also treated with appropriate isotype control antibodies (eBioscience). Stained cells were analyzed using flow cytometery. The data were analyzed by FlowJo software 7.6.1 (Tree star, Inc., San Carlos, CA, USA).

### Apoptosis assay

Forty-eight hours after transient transfection of KatoIII cells with miR-34a-5p mimic or negative control, the cells were collected and stained with Annexin V‑FITC/PI according to the manufacturer’s instructions (eBioscience; Cat. no. BMS500FI/100) to assay apoptosis. The apoptosis level of stably transfected cells was assessed using Annexin V‑PE/7-AAD (BioLegend; Cat. no. 640,908) in addition to Annexin V‑FITC/PI staining. Flow cytometry was performed to detect the apoptosis level of the transfected cells. The data was analyzed by FlowJo software 7.6.1 (Tree star, Inc., San Carlos, CA, USA).

### Cell proliferation assay with CFSE labeling

Cell proliferation assay was performed using CellTrace™ CFSE Cell Prolifereration Kit (Invitrogen, Cat.no: c34554) according to the manufacturer’s instructions. Briefly, the stably transfected cells were adjusted to a density of 8.5 × 10^5^ cells/ml and treated with CFSE at a final concentration of 25 µM in PBS. After incubation at 37 °C for 15 min, labeling was blocked by the addition of RPMI medium with 10% FBS. The cells were incubated for another 30 min. After washing, the collected cells were cultured in 4 flasks 25 cm^2^ for proliferation assessment at days 1–4. The fluorescence intensity was assayed each day by flow cytometry analysis. FlowJo software 7.6.1 (Tree star, Inc., San Carlos, CA, USA) was used to generate plots and data presentation.

### Statistical analysis

Statistical analysis was performed using GraphPad Prism^TM^ software version 6 (GraphPad Software Inc., CA, USA). Values are expressed as mean ± standard deviation (SD). The statistical significance of mean values between the two groups was analyzed by unpaired t test. Two‑tailed p-values less than 0.05 were considered statistically significant differences (*p < 0.05, **p < 0.01, ^**^p < 0.001).

## Results

### Expression of β-catenin and Notch1, two targets of miR-34a-5p, is deregulated in KatoIII cells

**Fig. 1:**
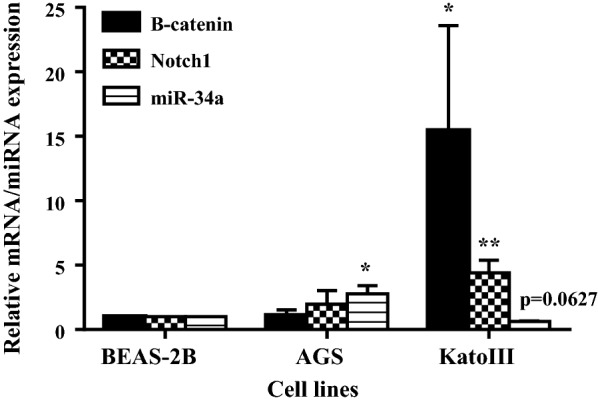
Expression levels of miR-34a-5p, β-catenin and Notch1 in gastric cancer cell lines (KatoIII and AGS) relative to a normal epithelial cell line (BEAS-2B). The assessment was performed using qRT-PCR. The statistical difference of gene expression level between each gastric cancer cell line and normal cell line was analyzed by unpaired t test. *p < 0.05; **p < 0.01

Using qRT-PCR analysis, the expression levels of miR-34a-5p and its two target genes, namely, β-catenin and Notch1 were evaluated in two GC cell lines, KatoIII and AGS, in comparison to normal epithelial cell line BEAS-2B. We found that the expression levels of two target genes of miR-34a-5p, namely, β-catenin and Notch1 within KatoIII cell line were considerably higher than those in normal epithelial cell line BEAS-2B (p < 0.05, and p < 0.01, respectively) as well as in AGS cell line. Furthermore, the result demonstrated that the miR-34a-5p expression level was lowest in the KatoIII cells (Fig. 1). Therefore, we chose KatoIII cell line for further examinations. The potential binding sites for miR-34a-5p in the 3′-UTR of β-catenin, Notch1, and CD44 mRNAs were showed in Additional file 1: Table S2.

### Transfection of miR-34a-5p mimic shows non‐specific effects on target gene expression in KatoIII cells

**Fig. 2:**
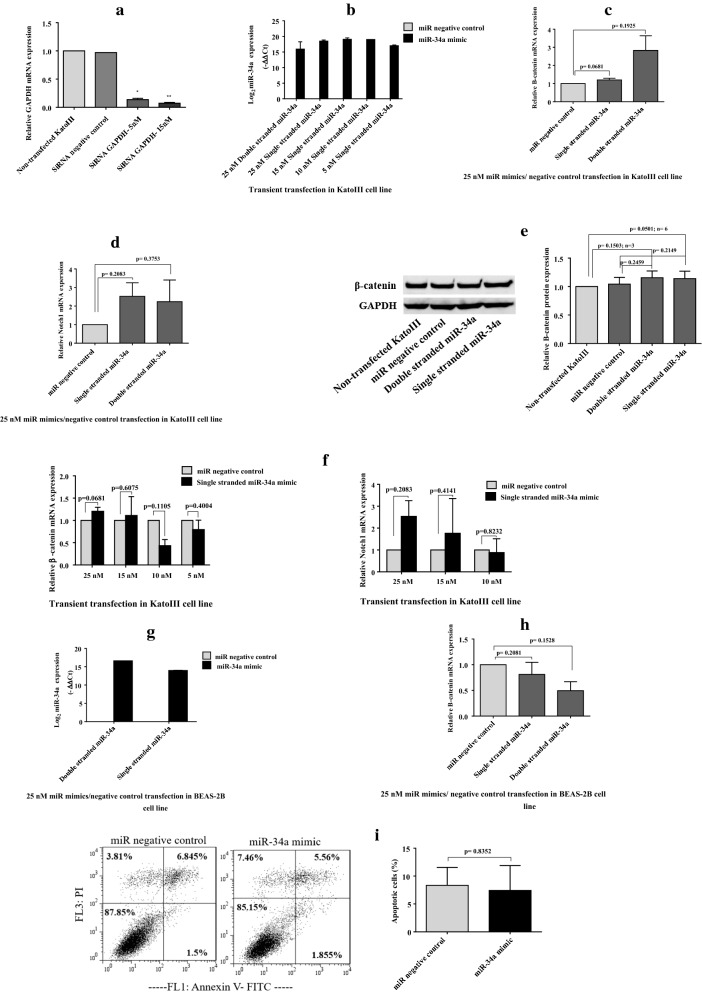
The effect of transient transfection of miR-34a-5p mimic. **a** GAPDH siRNA was transiently transfected into KatoIII cells and used as positive control for transient transfection. The GAPDH mRNA was quantified using qRT-PCR analysis and normalized against β- actin internal control. The results displayed a significant reduction in GAPDH mRNA relative to the controls. **b** Different concentrations of miR-34a-5p mimic were transiently transfected into KatoIII cells. The qRT-PCR analysis verified that the miR-34a-5p was overexpressed in transfected cells relative to the control. **c**, **d** Transfection of double- or single- stranded miR-34a-5p mimic at 25 nM into KatoIII cells did not reduce the mRNA levels of β-catenin and Notch1. But, higher levels of target mRNAs were observed based on qRT-PCR analysis. **e** Western blot analysis showed that transient transfection with 25 nM miR-34a-5p mimic did not decrease the β-catenin protein level, but led to increase the protein level; thus, the data were consistent with qRT-PCR. **f** Different concentrations of miR-34a-5p mimic, including 15 nM, 10 nM, and 5 nM, were also transfected into KatoIII cells. Quantification of target mRNAs, β-catenin and Notch1, using qRT-PCR showed that miR-34a-5p mimic had inhibitory effect on these two target genes expression at lower concentrations, 10 nM and 5 nM, although the changes were not statistically significant. **g**, **h** The effect of transiently transfected miR-34a-5p mimic at 25 nM on the β-catenin expression was evaluated in BEAS-2B cell line. Using qRT-PCR analysis, a remarkably reduced level of β-catenin mRNA was detected in BEAS-2B cell line. **i** The effects of transient transfection of miR-34a-5p mimic on the apoptosis rate was assessed using flow cytometry analysis. Compared with the control cells, significant change was no observed in the percentage of apoptotic cells following transfection of miR-34a-5p mimic at 25 nM. *p < 0.05; **p < 0.01

First we transiently transfected miR-34a-5p mimic into KatoIII cells. The qRT-PCR results showed that the miR-34a-5p was overexpressed in transfected cells relative to NC cells (transfected with negative control sequence) (Fig. 2b). We then investigated the effect of transiently transfected miR-34a-5p mimic on the target gene expression in KatoIII cell line. Upon transfection with 25 nM miR-34a-5p mimic (either double- or single- stranded sequences), the mRNA levels of two target genes were not reduced. Unexpectedly, the changes of target gene expression were toward higher expression (Fig. 2c, d). In addition, western blot analysis showed that transient transfection with 25 nM miR-34a-5p mimic did not lead to reduce β-catenin protein level (Fig. 2e); thus, the data were consistent with qRT-PCR.

Next, different concentrations of miR-34a-5p mimic, including 15 nM, 10 nM, and 5 nM, were also transfected into KatoIII cells and target gene mRNA was quantified by qRT-PCR. Data showed that the miR-34a-5p mimic had inhibitory effect on target gene expression at lower concentrations, 10 nM and 5 nM, although these changes were not statistically significant (Fig. 2f).

Moreover, the effect of transiently transfected miR-34a-5p mimic at 25 nM on the β-catenin expression was evaluated in BEAS-2B cell line. Contrary to what was observed for KatoIII cell line, a remarkably reduced level of β-catenin mRNA was observed in BEAS-2B cell line (Fig. 2g, h).

### Transfection of miR-34a-5p mimic does not induce apoptosis in KatoIII cells

The effect of miR-34a-5p mimic on the apoptosis of KatoIII cells was assessed using flow cytometry analysis. Compared with the NC cells, significant difference was no observed in the percentage of apoptotic cells following transfection of the miR-34a-5p mimic at 25 nM (Fig. 2i).

### Transfection of miR-34a-5p mimic has no effect on expression of putative GCSC markers

Putative GCSC markers in KatoIII cells were measured by flow cytometry.

**Fig. 3:**
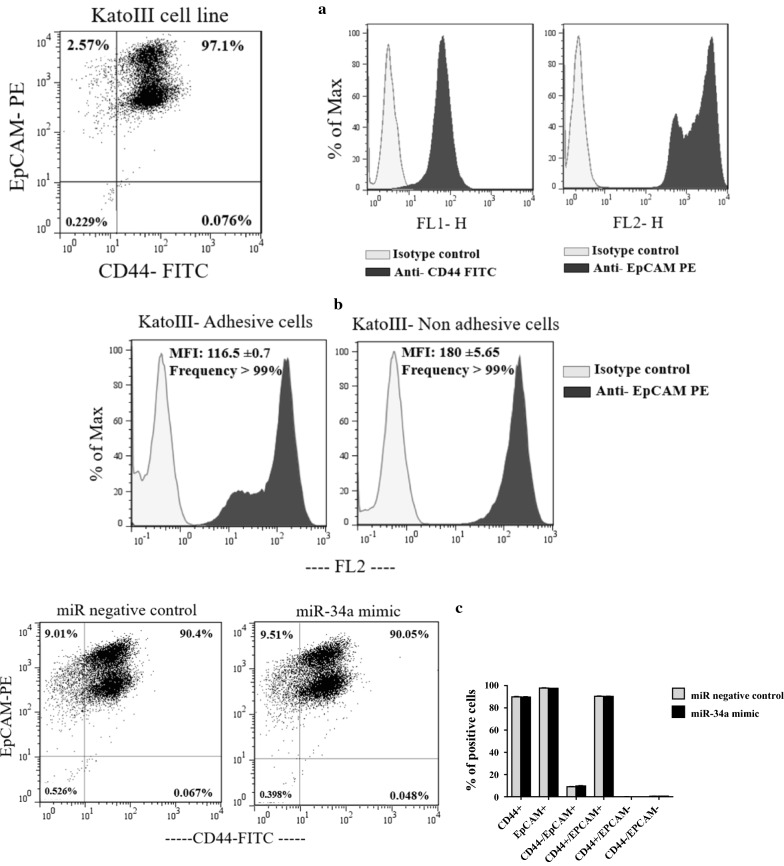
Expression levels of CD44 and EpCAM on KatoIII cells surface and the effect of transient transfection of miR-34a-5p mimic on these markers expression. **a** The flow cytometry analysis indicated that EpCAM and CD44 are expressed in almost all and ~ 97% of KatoIII cells, respectively. The expression of CD44 on KatoIII cells showed lower intensity than EpCAM expression. In addition, two sub-populations of EpCAM positive cells with different fluorescence intensity were observed in KatoIII cell line. **b** The frequency and MFI of EpCAM marker were compared between adhesive KatoIII cells dissociated from the flask using trypsin enzyme and non-adhesive KatoIII cells. Data showed that the use of trypsin enzyme for detachment of the adherent cells from the flask reduced the fluorescence intensity of EpCAM (116.5 ± 0.7), in comparison to the suspended cells (180 ± 5.65). But, the use of trypsin enzyme for detachment of the adherent cells had no effect on the frequency of EpCAM positive cells. **c** According to the flow cytometry analysis, transient transfection of the miR-34a-5p mimic at 25 nM had no effect on percentage of CD44 and EpCAM positive cells in KatoIII cells. MFI: Median Fluorescence Intensity

We first examined the expression level of CD44 and EpCAM in untreated KatoIII cells. The results showed that EpCAM and CD44 are expressed in almost all and ~ 97% of KatoIII cells, respectively. In addition, CD44 expression on KatoIII cells showed lower intensity than EpCAM expression. Furthermore, we found two sub-populations of EpCAM positive KatoIII cells with different fluorescence intensity (Fig. 3a). Given that KatoIII is a semi-adhesive cell line, we assumed that this observation could be attributed to the mentioned physiological property. In next experiment, we collected KatoIII cells adherent to the culture flask using trypsin enzyme (0.25%) as well as the suspended cells in the culture medium. These two cell fractions were then analyzed separately for EpCAM expression. The results showed that the use of trypsin enzyme for detachment of the adherent cells from the flask reduced the fluorescence intensity of EpCAM but did not affected on the frequency of EpCAM positive cells (respectively, 116.5 ± 0.7 and > 99%), in comparison to the non-adherent (suspended) cells (respectively, 180 ± 5.65 and > 99%). This was the reason why we did not focus on MFI (Median Fluorescence Intensity) change of EpCAM in experiments with KatoIII cell line. The results were given in Fig. 3b.

We next evaluated the effect of miR-34a-5p mimic on putative CSCs markers. According to the flow cytometry analysis, we found that transient transfection of miR-34a-5p mimic at 25 nM had no effect on percentage of CD44 and EpCAM positive cells in KatoIII. Furthermore, no change of MFI of CD44—previously verified as direct target of miR-34a-5p—was detected (Fig. 3c).

### Stable transfection of pre-mir-34a efficiently suppresses the target gene expression

**Fig. 4:**
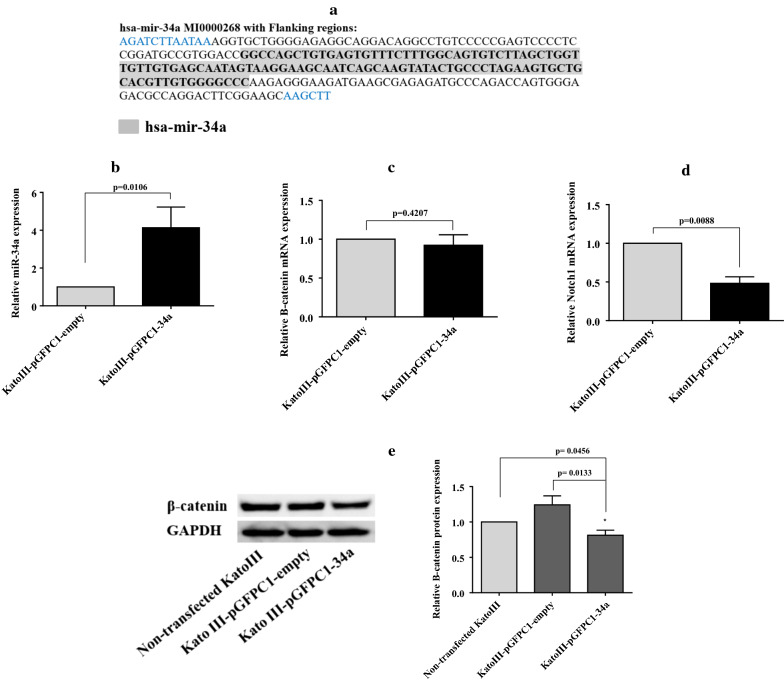
The effect of stable transfection of pre-mir-34a on target genes expression. **a** Sequence of DNA fragment, 248 base pairs in length, including pre-mir-34a (highlighted in grey) and flanking sequences was obtained from the human gene. The DNA fragment was cloned in the MCS between BglII and HindIII restriction sites. The RNA expression levels of miR-34a-5p, β-catenin and Notch1were quantified using qRT-PCR analysis. **b** The miR-34a-5p expression was significantly enhanced in KatoIII-pGFPC1-34a cells compared with the equivalent control cells, KatoIII-pGFPC1-empty (p < 0.05). **c** A non-significant mild reduction of β-catenin mRNA level was determined in KatoIII-pGFP-34a cells relative to the KatoIII-pGFPC1-empty cells. **d** The Notch1 mRNA level was significantly decreased in KatoIII-pGFP-34a cells relative to KatoIII-pGFPC1-empty cells (p < 0.01). **e** The expression level of β-catenin protein was detected using western blot analysis. The data demonstrated that the β-catenin protein expression was significantly reduced in KatoIII-pEGFPC1-34a cells relative to the control cells (p < 0.05)

When stable cell lines, named as KatoIII-pGFPC1-34a and KatoIII-pGFPC1-empty, were stablished, miR-34a-5p level was analyzed using qRT-PCR within the cells at different passages to verify the stable transfection efficiency. Compared with the equivalent control cells (KatoIII-pGFPC1-empty), miR-34a-5p expression was significantly enhanced in KatoIII-pGFPC1-34a cells (p < 0.05; Fig. 4b). Next, the expression level of Notch1 and β-catenin mRNAs was evaluated using qRT-PCR analysis. The result indicated that Notch1 mRNA level was significantly decreased in KatoIII-pGFP-34a cells relative to equivalent control, namely KatoIII-pGFP-empty cells (p < 0.01) (Fig. 4d). In spite of a non-significant marginal reduction of β-catenin mRNA level in KatoIII-pGFP-34a cells, a significant decrease of β-catenin protein level was revealed using western blot analysis (p < 0.05) (Fig. 4c, e). These results illustrated that the predominant effect of miR-34a-5p on β-catenin is exerted at the translation level.

### Stable transfection of pre-mir-34a in KatoIII cells significantly reduces CD44 + cells

**Fig. 5:**
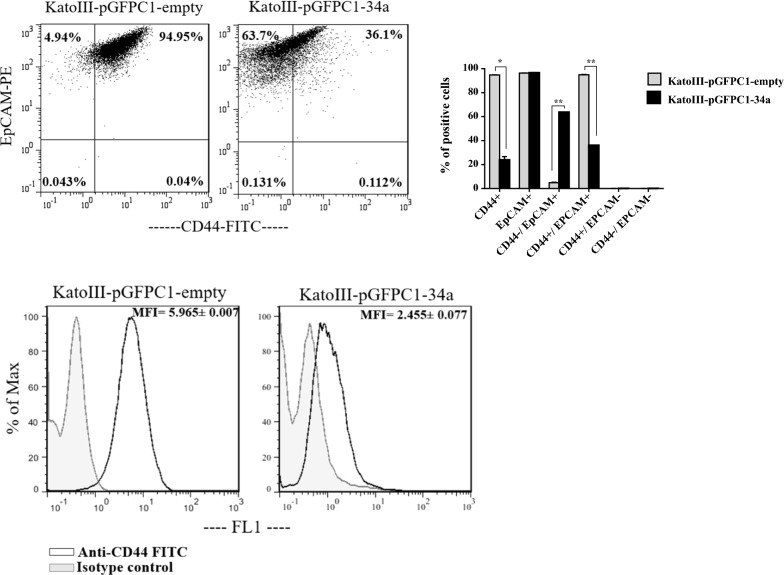
The effect of stable transfection of pre-mir-34a on the expression levels of CD44 and EpCAM markers. The flow cytometric detection illustrated that the percentage of CD44 + cells and the MFI of CD44 marker were significantly decreased in the KatoIII-pGFPC1-34a cells compared to the KatoIII-pGFPC1- empty cells. No difference was found in percentage of EpCAM + cells between these two stable cells. *p < 0.05; **p < 0.01. MFI: Median Fluorescence Intensity

Based on flow cytometric detection, the percentage of CD44 + cells in the KatoIII-pGFPC1-34a cells (< 40%) was significantly lower than that in the KatoIII-pGFPC1- empty cells (~ 95%) (p < 0.05). Furthermore, fluorescence intensity of CD44 marker was significantly decreased in KatoIII-pGFPC1-34a cells (2.455 ± 0.077) compared to the KatoIII-pGFPC1- empty cells (5.965 ± 0.007). Thus, transfection of mir-34a had remarkable effect on CD44 repression. Moreover, no difference was found in percentage of EpCAM + cells between these two stable cells. However, with reduction of CD44 + cells in KatoIII-pGFPC1-34a cells, the percentage of CD44 + EpCAM + population, assumed to be attributed to GCSCs, was also decreased (p < 0.01) (Fig. 5).

### Stable transfection of pre-mir-34a in KatoIII cells efficiently induces apoptosis

Because of the enhanced auto-fluorescence intensity of KatoIII-pGFPC1-34a specially in FL1 channel (unpublished data), we assayed apoptosis rate of the cells with FITC- conjugated Annexin V/PI, as well as PE-conjugated Annexin V/7AAD, to reach a reliable data.

**Fig. 6:**
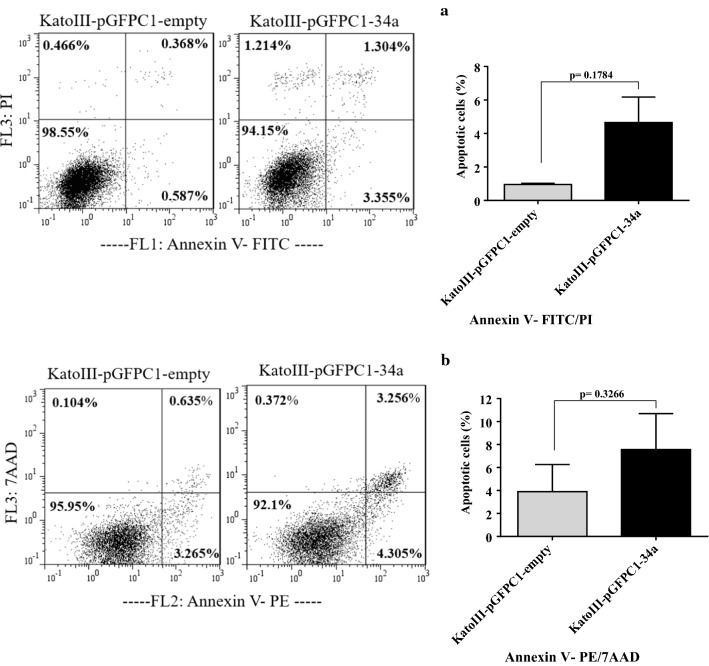
The effect of stable transfection of pre-mir-34a on the apoptosis rate. The flow cytometric assessments using FITC- conjugated Annexin V/PI (**a**) or PE-conjugated Annexin V/7AAD (**b**) demonstrated an increase of ~ 3.5% in apoptotic cells of KatoIII-pGFPC1-34a cells compared with KatoIII-pGFPC1-empty cells

Using flow cytometric assays, we detected an increase of ~ 3.5% in apoptotic cells of KatoIII-pGFPC1-34a cells compared with equivalent control. These results suggested that increased expression of mir-34a promoted apoptosis (Fig. 6a, b).

### Stably transfected KatoIII cells with pre-mir-34a show slow proliferation rate

**Fig. 7:**
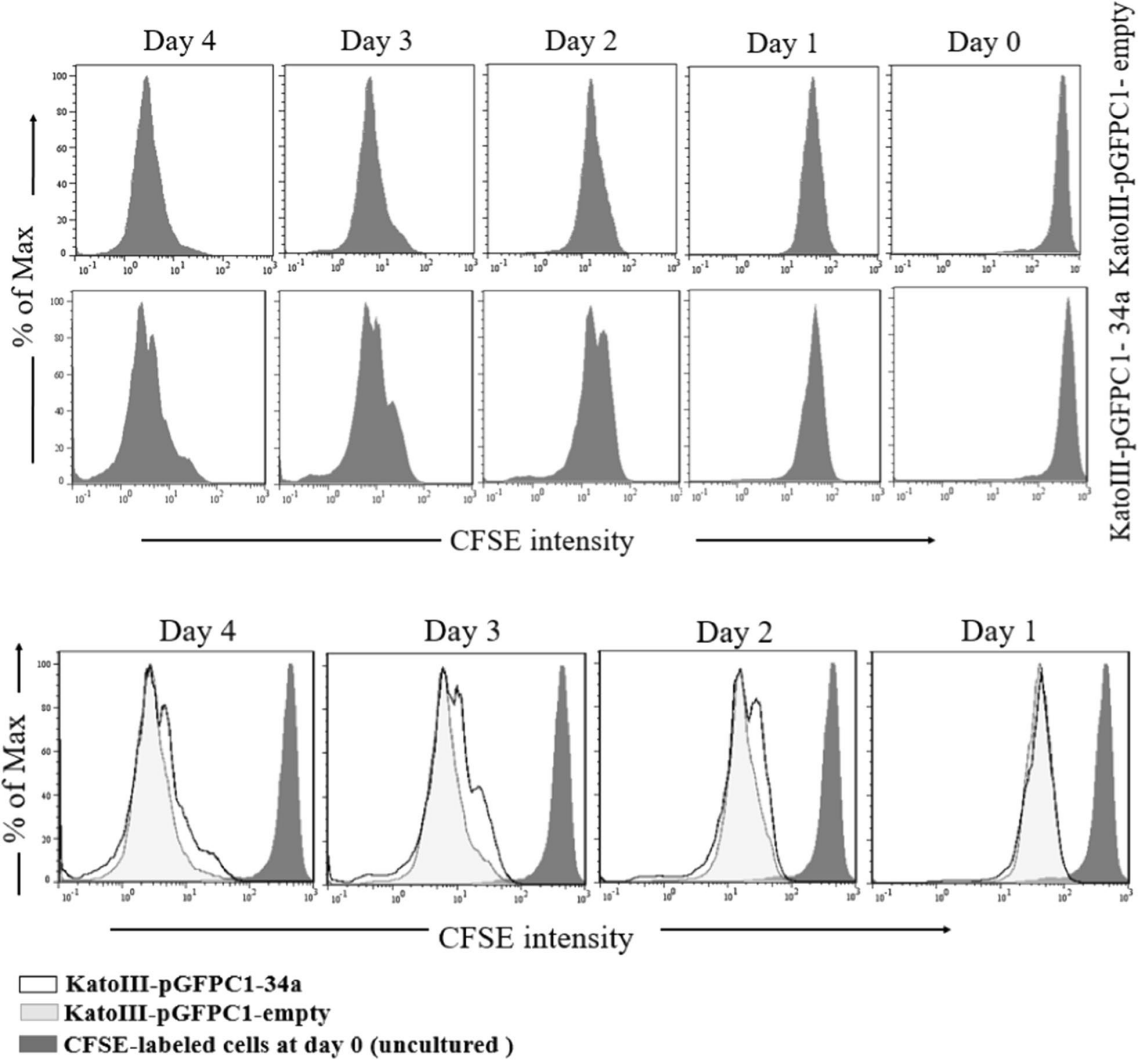
The effect of stable transfection of pre-mir-34a on the cell proliferation. The flow cytometric assessment using CFSE labeling demonstrated that total number of KatoIII-pGFPC1-34a and KatoIII-pGFPC1- empty cells were proliferated at day1, but KatoIII-pGFPC1-34a cells multiplied slowly during the 3 days in culture. Overlay plots were displayed at the bottom

The CFSE assay was used to investigate the effects of mir-34a on cell proliferation. Flow cytometric analysis demonstrated that total cells of KatoIII-pGFPC1-34a and KatoIII-pGFPC1- empty were proliferated at day1, but KatoIII-pGFPC1-34a cells multiplied slowly during the 3 days in culture (Fig. 7).

## Discussion

In the present study, we first transiently transfected 25 nM miR-34a-5p mimic into KatoIII cells to increase cellular concentration of miR-34a-5p. Following transfection, not only inhibition of two target genes expression, Notch1 and β-catenin, was observed, but a non-significant marginal increase of those was detected (Fig. 2). In addition, no changes were detected in the percentage of apoptotic cells as well as in the CD44 + EpCAM + cells after transient transfection of 25 nM miR-34a-5p mimic into KatoIII cells (Figs. 2i and 3c). Next, we stably transfected mir-34a (precursor) into KatoIII cells and examined the effects of enhanced expression of mir-34a on cellular physiology. Interestingly, stable transfection of mir-34a caused a significant reduction in expression of β-catenin protein and Notch1 mRNA (Fig. 4). Furthermore, lower level of CD44 expression, higher percentage of apoptotic cells and a slower proliferation rate were detected in KatoIII-pGFPC1-34a cells than KatoIII-pGFPC1-empty cells (Figs. 5, 6 and 7).

In previous reports, transient transfection of miR mimics or miR inhibitors were extensively used for restoration of aberrantly expressed miRNAs. In the following, we mentioned some studies suggesting that transiently transfected miR-34a-5p mimic is functional. Cao et al. study showed that transient transfection of miR-34a mimic in a GC cell line, HGC-27, inhibited cellular viability, proliferation and invasion levels. In addition, transiently transfected miR-34a mimic increased cellular apoptosis and negatively regulated survivin protein expression, as a target gene. But, there was no further information on the miR-34a mimic concentration used for transfection in this study [20]. In Peng et al. study, overexpression of miR-34a mimic in GC cell line, AGS, inhibited cell migration, invasion and proliferation. Meanwhile, Peng and co-workers reported that miR-34a mimic inhibits PDGFR-α/β protein (Platelet- derived growth factor receptor)—a direct target gene—without any information on the miR mimic concentration used for transfection [21]. In Zhu et al. study, transient transfection of 50 nM miR-34a mimic in SW620 colorectal cancer cells caused a considerable decrease in cell migration, invasion and proliferation rates, and a downregulation of vimentin and upregulation of EGR1 (early growth response protein 1) protein levels [22]. On the basis of many previous studies, commonly used transient transfection concentrations were within the rang 20–100 nM for cancer cells [22–24]. Thus, we first examined the efficiency of transient transfection of miR-34a-5p at 25 nM—one of the frequently used concentrations in previous studies—in KatoIII cells. In spite of an efficient transient transfection that was verified using qRT-PCR, we were unable to detect any expected functions of miR-34a-5p, as mentioned above. Meanwhile, we also observed this non-specific effect of miR-34a-5p at 15 nM on target gene expression (Fig. 2f). In the following sections we attempted to clarify the plausible reasons for ineffectiveness of transient transfection into KatoIII cells.

Consistent with our observation, limited evidence indicates that transiently transfected miR mimics may not function similarly to endogenous miRNAs. Jin et al. suggested that the supraphysiological expression levels of mature miRNAs, which resulted from transient transfection of miRNA mimics at high concentrations (25 and 100 nM), led to the accumulation of high molecular weight RNA species and non-specific changes in gene expression. While at low concentrations, transfected miRNAs failed to efficiently suppress target gene expression. In contrast, expression of the same miRNAs by lentiviral transduction, plasmid transfection, and endogenous overexpression caused less than 10-fold increase with no appearance of high molecular weight RNA species, that was sufficient to suppress target gene expression [25].

As we have aforementioned, in our work, DNA of mir-34a precursor was stably transfected into KatoIII cells to produce KatoIII-pGFPC1-34a cells. Thus, another possible reason for the different findings that were achieved in transiently and stably transfected KatoIII cells can be attributed to the limited evidences supporting a direct function of pri-/pre- miRNAs in target recognition and repression [10, 11]. Consistent with this view, in Liu et al. study, murine miRNA genes mir-181a-1 and mir-181c, which encode mature miRNAs with a single nucleotide difference, were reported to have distinct functions in early T cell development. Liu and co-workers found that the distinct activities of mir-181a-1 and mir-181c are largely determined by their unique pre-miRNA loop nucleotides. They further suggested that both the strength and the functional specificity of miRNA genes can be regulated by the pre-miRNA loop nucleotides [10]. Another study showed that loop nucleotides of human pri-let-7 has a direct regulatory function in repression of target mRNAs even in the presence of correctly processed pre- and mature miRNAs [11].

In the following, we discussed the findings obtained from the study on stably transfected KatoIII cells with plasmids, pGFPC1-34a and pGFPC1-empty as control.

The canonical Wnt/β-catenin signaling pathway is known as a key contributor in biology of CSCs [26]. The CSCs biomarkers, including c-myc, Nanog, Oct3/4, Sox2 and EpCAM are targets of this pathway [27]. Important role of the Wnt/β-catenin signaling pathway in self-renewal and maintenance of CSCs has been reported in various cancers, including hepatocellular carcinoma [28], nasopharyngeal carcinoma [27], and GC [29]. Importantly, two biomarkers that frequently reported in related to GCSCs, namely CD44 and EpCAM, have been found as transcriptional targets of Wnt/β-catenin signaling pathway [27, 28, 30]. Malanchi et al. found that genetic ablation of β-catenin in squamous cell carcinomas cell line (SCC13) caused to loss of CSCs and decreased xenograft tumor growth in nude mice [31].

In our study, β-catenin protein level, the percentage of CD44 + cells as well as CD44 expression intensity were significantly decreased in KatoIII-pGFPC1-34a cells relative to KatoIII-empty cells (Figs. 4e and 5). CD44 marker was previously proved as a direct target gene of miR-34a [32]. Since CD44 marker was also found as a transcriptional target of Wnt/β-catenin signaling pathway [26], thus, reduction of CD44 in our study can be occurred due to a direct inhibitory effect of miR-34a-5p as well as to a decreased level of β-catenin protein expression. In addition, previous studies showed that CD44 regulates Wnt signaling. In other words, up- or down-regulation of different isoforms of CD44, respectively, positively or negatively modulated Wnt activity [33]. For instance, Chang et al. reported that down-regulation of CD44 expression in K562 cells using transfection of CD44 shRNA decreased β-catenin expression and its subsequent accumulation in the nucleus [34].

Yamashita et al. reported that the destruction of β-catenin or inhibition of Tcf/ β-catenin complex formation decreased EpCAM expression in normal human hepatocytes and HCC cell lines [30]. Inconsistent with the above study, though repression of β-catenin protein was detected in our study, the percentage of EpCAM + cells was not reduced. However, we were unable to definitively assay of EpCAM expression intensity (or MFI) by flow cytometry analysis due to the destructive effect of trypsin enzyme—used for detachment of adherent cells from the culture flask—on EpCAM protein (Fig. 3b). It is worth noting that with reduction of CD44 + cells, the percentage of CD44 + EpCAM + cells—known to be enriched for GCSCs—was also decreased (Fig. 5).

We detected a significant repression of Notch1 mRNA in KatoIII-pGFPC1-34a cells compared to KatoIII-pGFPC1-empty cells. In previous studies, Notch1 and Notch2 (Notch signaling receptors) as well as JAG1 and DLL1 (Notch signaling ligands) were verified as direct targets of miR-34a [15]. Notch signaling pathway involves in various cellular processes, including proliferation, differentiation, apoptosis, adhesion, angiogenesis, and EMT (epithelial to mesenchymal transition), depending on the cell type. Notch family proteins act as receptors for extracellular signals and also function as transcription factors regulating the gene expression in the nucleus [35–37]. Increasing evidences suggest that Notch signaling may involve in self renewal regulation and maintenance of CSCs. As examples, Simmons et al. showed that inhibition of Notch1 in mouse mammary tumor cells reduced tumor sphere formation ability in vitro and caused mammary tumor regression in vivo—characteristics of CSCs —and prevented tumor recurrence [38]. Tatarek et al. found that Notch1 inhibition in mouse model of T-ALL (T-cell acute lymphoblastic leukemia) significantly reduced leukaemia CSCs and extended the animal survival [39].

In our study, in spite of a significant reduction in Notch1 mRNA level, a non-significant marginal reduction in β-catenin mRNA level was detected in KatoIII-pGFPC1-34a cells (Fig. 4c, d). Target mRNAs with higher affinity of complementary sites to cognate miRNAs commonly show greater sensitivity to post-transcriptional repression mediated by miRNAs [8]. The binding sites and minimum free energy attributed to miR-34a-5p and three target genes, β-catenin, Notch1 and CD44, retrieved from miRtarBase (http://mirtarbase.mbc.nctu.edu.tw), were presented as Additional file 1: Table S2.

We detected an increase of ~ 3.5% in percentage of apoptotic cells in KatoIII-pGFPC1-34a compared to the equivalent control cells, KatoIII-pGFPC1-empty (Fig. 6). Numerous studies demonstrated the miR-34a effect on apoptosis induction and proliferation inhibition [20, 40, 41]. Cao et al. demonstrated that transient transfection of miR-34a mimic into HGC-27, a GC cell line, promoted apoptosis to about 25% and decreased proliferation rates compared to the control cells [20]. Li et al. reported that transient transfection of miR-34a mimic into breast cancer cell lines, including MDA-MB-231 and MDA-MB-435 enhanced apoptosis to 23.1% and 18.8%, respectively, and suppressed proliferation compared to the control cells [40].

Several studies reported a mechanism dependent to p53 protein in induction or promotion of apoptosis by miR-34a. In Chang et al. study miR-34a mimic was transiently transfected into *P53* wild-type HCT116 colon cancer cell line (HCT116 *P53*^WT^) and an isogenic cell line that both alleles of *p53* were inactivated in which (HCT116 *P53*^−/−^). Relative to equivalent controls, transfection of miR-34a in HCT116 *P53*^WT^ cells potently enhanced apoptotic cell death (17.6%). Whilst, apoptosis rate was substantially low in HCT116 *P53*^−/−^ cells (5.2%), though not completely removed [41]. Moreover, Gao et al. found that ectopic expression of miR-34a-5p in HCT116 *P53*^WT^ cells remarkably suppressed cell growth, migration, invasion and metastasis. Furthermore, miR-34a-5p induced apoptosis and cell cycle arrest at G1 phase in HCT116 *P53*^WT^ cells, but not in HCT116 *P53*^−/−^ cells. Also, miR-34a-5p inhibited the growth of HCT116 *P53*^WT^ cells in vivo, while it had no effect on HCT116 *P53*^*−/−*^ xenograft, implying that the growth suppressive effect by miR-34a-5p was dependent on p53 [42]. Thus, the induction of apoptosis after transfection of miR-34a may exert in a p53-dependent manner.

The KatoIII cell line that was used in our study, is a p53-null GC cell line [43]. Although an enhanced apoptosis level of ~ 3.5% in KatoIII-pGFPC1-34a cells can be considerable, the reason of why further enhancement in cell apoptosis was not obtained, may be that KatoIII cells are deficient in p53 factor. Because p53 is frequently absent in human cancers [41], this issue challenges the potential use of miR-34a in treatment of patients.

Notwithstanding p53 acts as an important factor in activation of miR-34a transcription [41], however, we detected significant increase of miR-34a-5p in stably transfected KatoIII cells. This finding provides an evidence implying the involvement of other plausible pathway(s) in transcription of miR-34a.

In another phase of the present study an attempt was made to investigate the tumorigenic ability of stably transfected KatoIII cells. For this purpose, KatoIII-pGFPC1-34a, KatoIII-pGFPC1-empty and non-transfected KatoIII cells were subcutaneously injected in C57 nude mice. However, gastric tumor xenograft was not formed even in non-transfected KatoIII cells injection (data not shown). With regard to tumorigenic ability of KatoIII cells in nude mice, contradictory results have been reported. While several groups demonstrated the tumorigenic capacity of KatoIII cells in nude mice [43–46], others failed to develop the tumor in nude mice [47, 48]. We do not know a precise reason for the inconsistent reports attributed to the KatoIII tumorigenicity in nude mice. However, tumorigenic assessment of KatoIII cells in other immunodeficient animal models such as hamster may be useful in future studies.

## Conclusions

Since key genes involved in Wnt/ β-catenin signaling such as Wnt1, LRP6, β-catenin, TCF/LEF and CD44 as well as in Notch signaling pathway such as Notch1, Notch2, JAG1 and DLL1 were known as direct target genes of miR-34a-5p [15] and regarding to the important roles of these signaling pathways in cancer stemness maintenance, miR-34a-5p could be a pivotal molecule in suppression of CSCs. However, our findings raised two important questions; Which one(s) of the RNA species generated from miRNA genes, namely pri-/ pre-miRNAs or mature miRNA, have more robust activity in regulation of target genes? What amount (or dose) of miR mimics should be used in various treatments? The latter is particularly important because the effective dose may be different for different cancers, different subtypes of a cancer or even for each individual patient, mainly due to existing molecular heterogeneity. Our study revealed that the effect of miR mimic in target gene repression can be dependent to its concentration as well as to the cell type. Meanwhile, use of a tumor suppressor miR mimic in unsuitable concentration may exhibit contrary effects and activate the expression of oncogenic target genes. To overcome this problem, we speculate that use of a strategy for biogenesis and bioprocessing of miRNAs into deficient cells, similar to endogenous miRNAs, is more beneficial than use of synthetic mature miRNAs.

Definitively addressing to the above mentioned issues through further prospective studies will greatly improve our understanding of the molecular basis of miRNAs function and help to the development of more effective therapeutic strategies in cancer treatment.

## Supplementary Information

**Additional file 1. Table S1.** Specific reverse transcription (RT) and quantitative real time PCR primers. **Table S2.** Potential binding sites of miR-34a-5p in the 3ʹ UTR of target mRNAs, including β-catenin, Notch1 and CD44, as presented by miRtarBase (http://mirtarbase.mbc.nctu.edu.tw).

## Data Availability

All data generated during the study are presented in this article. Additional data may be found in the online version of this article.

## Abbreviations

GC: Gastric cancer
3ʹ-UTR: 3ʹ-untranslated region
miRNAs/miRs: microRNAs
qRT-PCR: Quantitative real time polymerase chain reaction
GFP: Green fluorescence protein
