# Supplementary material for: miR-34a mimic or pre-mir-34a, which is the better option for cancer therapy? KatoIII as a model to study miRNA action in human gastric cancer cells
Source: Cancer Cell Int. 2021 Mar 19;21:178. doi: 10.1186/s12935-021-01872-5 (PMC7980621; doi:10.1186/s12935-021-01872-5)
Supplement: Supplementary file 1 — Additional file 1. Table S1. Specific reverse transcription (RT) and quantitative real time PCR primers. Table S2. Potential binding sites of miR-34a-5p in the 3ʹ UTR of target mRNAs, including β-catenin, Notch1 and CD44, as presented by miRtarBase (http://mirtarbase.mbc.nctu.edu.tw). [file 12935_2021_1872_MOESM1_ESM.docx]

**miR-34a mimic or pre- mir-34a, which is the better option for cancer therapy? KatoIII as a model to study miRNA action in human gastric cancer cells**

**Narjes Jafari^1^, Saeid Abediankenari^1,2*^, Hadi Hossein-Nataj^2^**

^1^ Immunogenetics Research Center, Faculty of Medicine, Mazandaran University of Medical Sciences, Sari, Iran

^2^ Department of Immunology, Faculty of Medicine, Mazandaran University of Medical Science, Sari, Iran

* Corresponding author: [abedianlab@yahoo.co.uk](mailto:abedianlab@yahoo.co.uk)

**Supplementary Tables:**

Table 1. Specific reverse transcription (RT) and quantitative real time PCR primers

| Sequence (5ʹ-3ʹ) | Primer |
| --- | --- |
| GTCGTATCCAGTGCAGGGTCCGAGGTATTCGCACTGGATACGACACAACC | miR-34a stem-loop (RT) |
| GAATTTGCGTGTCATCCTTG | U6 specific (RT) |
| F: GGGATGGCAGTGTCTTAGC  Universal R: GTGCAGGGTCCGAGGT | miR-34a-5p |
| F: GCTTCGGCAGCACATATACTAAAAT  R: CGCTTCACGAATTTGCGTGTCAT | U6 |
| F: CAATGTGGATGCCGCAGTTGTG  R: CAGCACCTTGGCGGTCTCGTA | Notch1 |
| F: GATACCTCCCAAGTCCTGTATGAG  R: GCATCAAACTGTGTAGATGGGATC | β- catenin |
| F: CATGAGAAGTATGACAACAGCCT  R: AGTCCTTCCACGATACCAAAGT | GAPDH |
| F: GGCACCCAGCACAATGAAG  R: GCCGATCCACACGGAGTACT | β- actin |

F, forward; R, reverse

Table 2. Potential binding sites of miR-34a-5p in the 3ʹ UTR of target mRNAs, including β-catenin, Notch1 and CD44, as presented by miRtarBase (http://mirtarbase.mbc.nctu.edu.tw).

| MFE^*^ | Score | Position in 3ʹUTR | Duplex structure | Target gene |
| --- | --- | --- | --- | --- |
| -11.70 | 124.00 | 698-723 | miRNA 3' uguuggUCGAU- UC - - - UGUGACGGu 5'  \| \| : \| \| \| \| \| : \| \| \| \| \| :  Target 5' gtgttaAGTTATAGTGAATACTGCTa 3' | **CTNNB1**  **(NM_001904)** |
| -9.50 | 119.00 | 41-61 | miRNA 3' ugUUGGUCGAUUCUGUGACGGu 5'  \| \| : \| \| \| \| \| \| \| \| \| \|  Target 5 ' taAAATA - CTTTTACTCTGCCt 3' |  |
| -8.82 | 111.00 | 602-623 | miRNA 3' uguUGGUCGAUUCUGUGACGGu 5'  \| : \| \| : \| \| : : \| \| \| \| \|  Target 5' aatATCTGTAATGGTACTGACt 3' |  |
| -19.50 | 160.00 | 895-916 | miRNA 3' uguuggucgaUUCUGUGACGGu 5'  \| \| \| \| \| \| \| \| \| \| \|  Target 5' tttctggggaAAGACACTGCCt 3' | **Notch1**  **(NM_017617)** |
| -15.00 | 154.00 | 168-187 | miRNA 3' uguUG-GUCGAUUCUGUGACGGu 5'  \| \| \| \| \| \| \| \| \| \| \| \| \| \| \|  Target 5 ' tttACACAG--AA-ACACTGCCt 3' |  |
| -16.30 | 129.00 | 1369-1392 | miRNA 3' uguUGGUCGAUUC--UGUGACGGu 5'  \| \| : : \| \| \| \| : \| \| \| \| \|  Target 5' tgcACTGGGGACGCTCCGCTGCCg 3' |  |
| -21.20 | 139.00 | 381-403 | miRNA 3' uguUGGUCGAUU-CUGUGACGGu 5'  \| \| \| \| \| \| \| \| \| \| \| \| \| : \| \| \|  Target 5' cccACCAGCTAAGGACATTTCCc 3 | **CD44**  **(NM_001001392)** |
| -13.80 | 139.00 | 2342-2362 | miRNA 3' ugUUGGUCGAUUCUGUGACGGu 5'  \| \| \| \| : \| : \| \| \| : \| \| \| \|  Target 5 ' caAACC-CTTGCAACATTGCCt 3' |  |
| -14.50 | 136.00 | 768-783 | miRNA 3' ugUUGGUCGAUUCUGUGACGGu 5'  \| \| \| \| \| \| \| \| \| \| \| : \|  Target 5' taAACCAG------CACTGTCt 3' |  |

^*^ MFE: Minimum free energy.
